# FACTORS CONTRIBUTING TO SOCIAL ISOLATION AMONG OLDER RURAL RESIDENTS

**DOI:** 10.1093/geroni/igz038.1297

**Published:** 2019-11-08

**Authors:** Cassandra Cantave Burton

**Affiliations:** AARP, Washington, D.C, United States

## Abstract

AARP research finds one-third of adults age 45 and older consider themselves lonely. Analysis from the National Survey of Adults Age 45-plus on Loneliness and Social Connections will be presented. Findings indicate that sixty-one percent of respondents who have never spoken to a neighbor are lonely, compared with 33 percent who have spoken to a neighbor. Individuals earning less than $25,000, caregivers and LGBTQ people are more likely to be lonely. Moreover, the structure of one’s community also plays an important role in predicting loneliness and was significantly related to a person feeling lonely.

